# A Novel Protective Vaccine Antigen from the Core *Escherichia coli* Genome

**DOI:** 10.1128/mSphere.00326-16

**Published:** 2016-11-23

**Authors:** Danilo G. Moriel, Lendl Tan, Kelvin G. K. Goh, Minh-Duy Phan, Deepak S. Ipe, Alvin W. Lo, Kate M. Peters, Glen C. Ulett, Scott A. Beatson, Mark A. Schembri

**Affiliations:** a School of Chemistry and Molecular Biosciences, the University of Queensland, Queensland, Brisbane, Australia; b Australian Infectious Diseases Research Centre, the University of Queensland, Queensland, Brisbane, Australia; c School of Medical Sciences and Menzies Health Institute Queensland, Griffith University, Gold Coast, Queensland, Australia; Swiss Federal Institute of Technology Lausanne

**Keywords:** *Escherichia coli*, vaccines, virulence factors

## Abstract

*Escherichia coli* is a versatile pathogen capable of causing intestinal and extraintestinal infections that result in a huge burden of global human disease. The diversity of *E. coli* is reflected by its multiple different pathotypes and mosaic genome composition. *E. coli* strains are also a major driver of antibiotic resistance, emphasizing the urgent need for new treatment and prevention measures. Here, we used a large data set comprising 1,700 draft and complete genomes to define the core and accessory genome of *E. coli* and demonstrated the overlapping relationship between strains from different pathotypes. In combination with proteomic investigation, this analysis revealed core genes that encode surface-exposed or secreted proteins that represent potential broad-coverage vaccine antigens. One of these antigens, YncE, was characterized as a conserved immunogenic antigen able to protect against acute systemic infection in mice after vaccination. Overall, this work provides a genomic blueprint for future analyses of conserved and accessory *E. coli* genes. The work also identified YncE as a novel antigen that could be exploited in the development of a vaccine against all pathogenic *E. coli* strains—an important direction given the high global incidence of infections caused by multidrug-resistant strains for which there are few effective antibiotics.

**IMPORTANCE** *E. coli* is a multifaceted pathogen of major significance to global human health and an important contributor to increasing antibiotic resistance. Given the paucity of therapies still effective against multidrug-resistant pathogenic *E. coli* strains, novel treatment and prevention strategies are urgently required. In this study, we defined the core and accessory components of the *E. coli* genome by examining a large collection of draft and completely sequenced strains available from public databases. This data set was mined by employing a reverse-vaccinology approach in combination with proteomics to identify putative broadly protective vaccine antigens. One such antigen was identified that was highly immunogenic and induced protection in a mouse model of bacteremia. Overall, our study provides a genomic and proteomic framework for the selection of novel vaccine antigens that could mediate broad protection against pathogenic *E. coli*.

## Abstract (precis)

*E. coli* is a multifaceted pathogen of major significance to global human health and an important contributor to increasing antibiotic resistance. Given the paucity of therapies still effective against multidrug-resistant pathogenic *E. coli* strains, novel treatment and prevention strategies are urgently required. In this study, we defined the core and accessory components of the *E. coli* genome by examining a large collection of draft and completely sequenced strains available from public databases. This data set was mined by employing a reverse-vaccinology approach in combination with proteomics to identify putative broadly protective vaccine antigens. One such antigen was identified that was highly immunogenic and induced protection in a mouse model of bacteremia. Overall, our study provides a genomic and proteomic framework for the selection of novel vaccine antigens that could mediate broad protection against pathogenic *E. coli*.

## INTRODUCTION

*Escherichia coli* is a bacterium that exists in many different guises. On one hand, it is a common commensal organism of the human small intestine. On the other, it is a rapidly evolving pathogen that is able to acquire and combine different genetic elements into novel and complex gene repertoires. The latter has led to the evolution of *E. coli* as a multifaceted pathogen, highlighted by aggressive disease outbreaks (1) and the emergence of multidrug-resistant (MDR) lineages (2, 3).

*E. coli* can be classified into different pathotypes according to a common set of virulence factors and specific clinical manifestations (4). Despite these phenotypic associations, strains from a single pathotype are not restricted to one phylogroup; such strains can share the same genomic profile with other pathotypes (5) and be distributed over the entire span of the *E. coli* phylogenetic diversity (6, 7). These observations indicate a common evolutionary origin and divergence into different pathotypes as a result of the independent acquisition of specific virulence genes via multiple events of horizontal gene transfer (8).

The 2011 *E. coli* O104:H4 German outbreak provided a new perspective to our understanding of evolution and genome plasticity in the species. The outbreak strain had acquired key virulence genes from two different *E. coli* pathotypes (enteroaggregative *E. coli* [EAEC] and enterohemorrhagic *E. coli* [EHEC]) and, combined with genes encoding resistance to antibiotics, emerged as a highly virulent lineage that infected nearly 4,000 people and caused 54 deaths (9). Since this outbreak, it has been proposed that targeting of accessory components encoded by the *E. coli* genome may be insufficient to prevent the emergence of new pathogenic lineages, and broader strategies directed against conserved features of all strains may be more effective (10).

*E. coli* represents one of the most sequenced microorganisms in public databases, with draft and complete genome sequences available for strains isolated from different hosts, disease associations, and geographic locations. In this study, we took advantage of this large genome sequence resource to define core and accessory *E. coli* genes. Furthermore, we used the resultant data to better understand the structural phylogeny of *E. coli* and identified YncE as a highly conserved vaccine antigen that is protective against acute *E. coli* bacteremia.

## RESULTS

### EcoDS: a large data set of *E. coli* genome sequenced strains.

An *E. coli* data set (EcoDS) was generated from 1,700 genome sequences available on the NCBI public database. EcoDS contained 62 complete and 1,638 draft genome sequences and represented a highly diverse collection of *E. coli* strains (Table 1; see Fig. S1 in the supplemental material). Information regarding the source, disease, origin, or year of isolation was available for approximately 69% (*n* = 1,174) of strains. Analysis of this strain subset revealed the most common sources as human (*n* = 747) or cattle (*n* = 76) and the most common disease associations as bacteremia (*n* = 222) or diarrhea (*n* = 114). The majority of strains originated from North America (*n* = 327) or Asia (*n* = 265) (Table 1). Overall, EcoDS is represented by commensal *E. coli* strains as well as strains from all known *E. coli* pathotypes. This includes the intestinal pathotypes EAEC, EHEC, enteroinvasive *E. coli* (EIEC), adherent-invasive *E. coli* (AIEC), enteropathogenic *E. coli* (EPEC), enterotoxigenic *E. coli* (ETEC), and the recently emerged enteroaggregative and hemorrhagic *E. coli* (EAHEC) pathotype, as well as extraintestinal pathotypes comprising avian-pathogenic *E. coli* (APEC), neonatal meningitis *E. coli* (NMEC), and uropathogenic *E. coli* (UPEC).

10.1128/mSphere.00326-16.1 Figure S1  Classification of the database according to the region and year of isolation, source, and disease. Download Figure S1, TIF file, 2.7 MB. Copyright © 2016 Moriel et al. 2016 Moriel et al. This content is distributed under the terms of the Creative Commons Attribution 4.0 International license.

**TABLE 1:** Distribution of strains in EcoDS according to source, disease, year, and place of isolation

Source	%	Disease^a^	%	Yr	%	Place	%
Bat	0.1	Asymptomatic	0.8	<1940s	0.1	Africa	0.8
Buffalo	0.1	Bacteremia	13.1	1950s	0.5	Asia	15.6
Cow	4.5	Bacteriuria	0.1	1960s	0.6	Europe	7.3
Dog	0.2	Crohn’s disease	0.2	1970s	0.7	North America	19.4
Environment	0.9	Diarrhea	6.7	1980s	2.2	South America	1.5
Fish	0.1	HUS	1.1	1990s	5.5	Oceania	0.8
Food	1.0	Mastitis	0.4	2000s	12.2	Unknown	54.6
Goat	0.1	Meningitis	0.2	2010s	9.6		
Horse	0.1	Omphalitis	0.1	Unknown	68.6		
Human	43.9	Peritonitis	0.1				
Marsupial	0.9	RTI	0.1				
Mouse	0.1	Septicemia	0.2				
Pig	1.1	UTI	2.1				
Poultry	0.8	Unknown	74.8				
Rabbit	0.2						
Reptile	0.1						
Sheep	0.1						
Wild bird	0.6						
Unknown	45.1						

a HUS, hemolytic-uremic syndrome; RTI, respiratory tract infections; UTI, urinary tract infections.

### Phylogenetic and pathotype relationship of *E. coli* strains in EcoDS.

**FIG 1:**
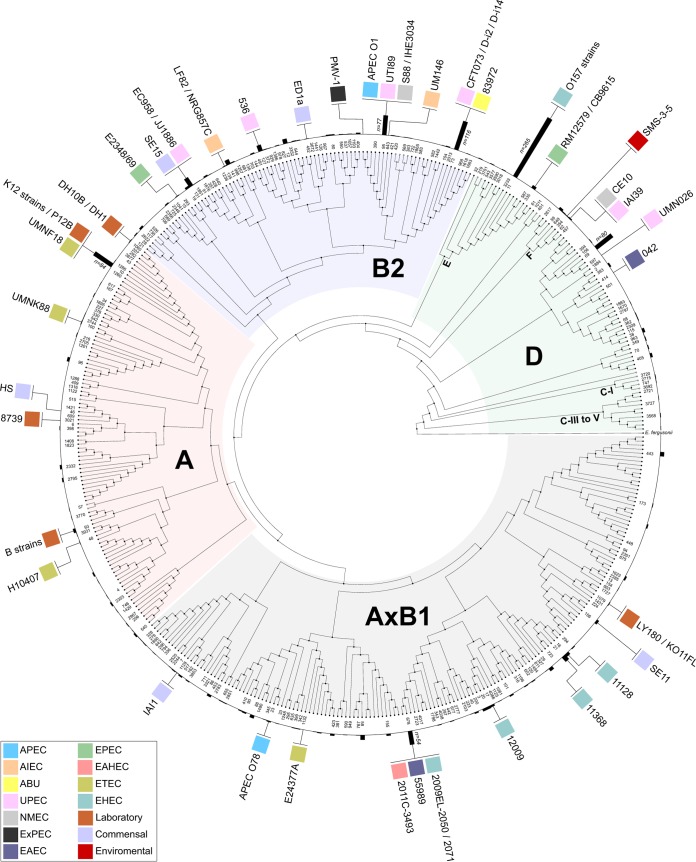
Phylogeny of *E. coli* strains in EcoDS. Phylogenetic tree demonstrating the relationship of MLSTs in EcoDS. Major phylogroups A, B1, B2, and D, as well as minor phylogroups E, F, C-I, and C-III to -V, are indicated. Completely sequenced strains are indicated according to their pathotype association (see the inset for the color-coded legend): (i) O157 strains, EDL933, Sakai, TW14359, EC4115, and Xuzhou21; (ii) K-12 strains, MC4100, MDS42, W3110, MG1655, c321.deltaA, and BW2952; and (iii) B strains, REL606, BL21(DE3), and BL21-Gold(DE3)(pLysS) AG. Histograms represent the number of genomes in the respective ST within EcoDS. STs represented by more than 50 genomes are indicated. Open circles represent STs that belong to a different phylogroup.

Phylogenetic group determination and multilocus sequence typing (MLST) were performed to characterize the relationship of all of the strains in EcoDS. Using this combined analysis, a phylogenetic tree was constructed based on 435 unique MLSTs identified in EcoDS (Fig. 1). This strategy confirmed the strong correlation between *E. coli* STs and the established multilocus enzyme electrophoresis (MLEE)-based *E. coli* phylogeny (11) and described a comprehensive distribution of STs within the major phylogroups in EcoDS (Fig. 1).

To establish a correlation between the *E. coli* phylogeny and pathotypes, the major phylogroups were analyzed in greater detail by a comparison of multiple factors, including year of isolation, origin, source, and disease association (Table 1). No clear geographic, host, or disease-specific component correlated with this clonal phylogenetic framework, highlighting a limitation for analysis of large collections with incomplete clinical sampling information (12).

EcoDS contained a highly diverse combination of STs and pathotypes (Fig. 1). The composition of phylogroup A includes K-12 (MG1655, W3110, MC4100, MDS42, c321.deltaA, BW2952, and DH10B) and B [BL21(DE3) and BL21-Gold(DE3)(pLysS) AG] strains, as well as most of the complete ETEC genome sequences and the commensal strain HS. The K-12 and UMNF18 strains belong to ST10, which is also represented by strains from UPEC (ATCC 23506), EAEC (C43/90), and APEC (S17) pathotypes. Phylogroup AxB1 is a hybrid group that comprehends strains from phylogroups A and B1. It contains the ST678 EAHEC strains involved in the 2011 German outbreak (1, 13–15) and strains from progenitor EHEC and EAEC STs that contributed to the emergence of this highly virulent lineage (e.g., the EAEC strain 55989 and the EHEC strains 2009EL-2050 and 2009EL-2071). Interestingly, phylogroup AxB1 is also represented by ST675, recently described as containing EHEC strains involved in UTI cases (16). Moreover, this group also contains nonpathogenic strains and strains from APEC and ETEC pathotypes. Phylogroup B2 contains a large number of strains from ST73 (*n* = 116) and the recently emerged and globally disseminated MDR ST131 clonal lineage (*n* = 38) (17, 18). This phylogroup predominantly contains strains associated with extraintestinal human infections, although strains from AIEC and EPEC pathotypes are also present. Phylogroup D is represented by the clonal lineages including subgroup E, the previously described subgroup F (6), and the recently identified cryptic lineages referred to as C-I to C-V (19, 20). Phylogroup E is the least diverse group and is a clonal lineage predominantly represented by EHEC and EPEC strains belonging to ST11 (235/272 strains) and from O157 and O55 serogroups. Phylogroup D also comprehends the subgroup F, which is represented by the environmental isolate SMS-3-5, the NMEC strain ce10, and the UPEC strain IAI39. The cryptic lineages C-I to C-V include EHEC, ETEC, commensal strains, and the environmental isolates previously described (19, 20). Moreover, phylogroup D is also represented by strains from ST69 (n = 80), which includes the UPEC reference strain UMN026 and EAEC strain 42. Together, our analysis shows that strains from the same phylogroup, and even from the same ST, can be involved in different clinical manifestations and belong to different pathotypes.

### Determination of the *E. coli* core and accessory genome.

The majority of genomes within EcoDS comprised draft genome sequences, and we predicted that they would vary significantly in quality and coverage. Thus, we devised a strategy based on the prevalence of *E. coli* essential genes to remove genome sequences with poor or low coverage. First, we used two recent studies (21, 22) to define a set of 362 *E. coli* essential genes (see Data Set S1 in the supplemental material). We then screened for the prevalence of these essential genes in the 62 complete genomes, which should represent the best quality genome sequences in EcoDS. In total, 318 genes were present in all 62 completely sequenced strains. These 318 genes were therefore used to filter the 1,700 genomes in EcoDS, and our analysis revealed a prevalence of 99.64% ± 3.0% essential genes per strain (which allows a mean tolerance of one missing essential gene per strain). Therefore, strains missing more than one essential gene were discarded (*n* = 144 [see Data Set S2 in the supplemental material]), leaving a total of 1,556 strains in EcoDS. The least prevalent essential gene in EcoDS was *b3119* (*tdcR*), which was present in 99% (*n* = 1,541) of strains (see Data Set S3 in the supplemental material).

10.1128/mSphere.00326-16.3 Data Set S1  *E. coli* K-12 essential genes for growth in LB and their prevalence in the 62 *E. coli* complete genome sequences. Download Data Set S1, XLSX file, 0.1 MB. Copyright © 2016 Moriel et al. 2016 Moriel et al. This content is distributed under the terms of the Creative Commons Attribution 4.0 International license.

10.1128/mSphere.00326-16.4 Data Set S2  The 144 *E. coli* genome sequences missing more than one essential gene. Download Data Set S2, XLSX file, 0.1 MB. Copyright © 2016 Moriel et al. 2016 Moriel et al. This content is distributed under the terms of the Creative Commons Attribution 4.0 International license.

10.1128/mSphere.00326-16.5 Data Set S3  Prevalence of the 318 essential genes in the remaining 1,556 genome sequences (EcoDS). Download Data Set S3, XLSX file, 0.1 MB. Copyright © 2016 Moriel et al. 2016 Moriel et al. This content is distributed under the terms of the Creative Commons Attribution 4.0 International license.

**FIG 2:**
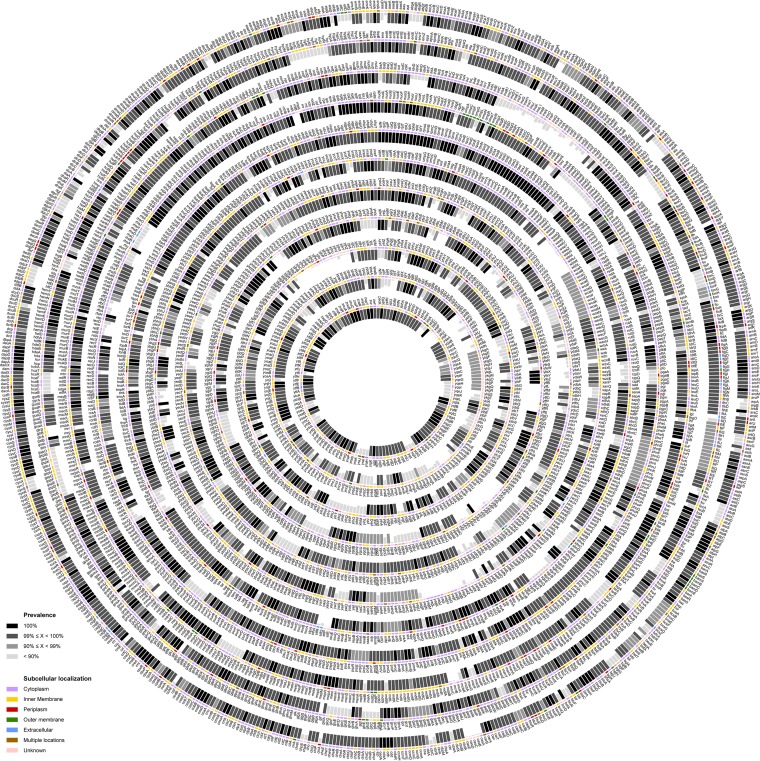
The *E. coli* core genome. The distribution of MG1655 genes among strains in EcoDS is represented by histograms and color coded according to prevalence and subcellular localization (predicted by PSORTb). Only the 4,319 unique genes in the MG1655 genome are shown.

We used the 1,556 genome sequences in EcoDS to define the conserved set of *E. coli* genes. To enable this analysis, we used the well-characterized and best-annotated K-12 strain MG1655 as a reference (23), and based on the essential gene data, a cutoff value for gene prevalence was set at 99%. Pairwise comparison of the 4,319 open reading frames (ORFs) defined in MG1655 with the genome sequence of the 1,556 strains in EcoDS led to the identification of 3,042 genes present in more than 99% of strains (protein identity of >75% over a 75% sequence overlap), of which 1,037 genes were present in 100% of the strains. These 3,042 genes define a conserved subset of *E. coli* genes in the 1,556 strains that make up EcoDS (Fig. 2; see Data Set S4 in the supplemental material).

10.1128/mSphere.00326-16.6 Data Set S4  Prevalence of 4,319 genes in EcoDS. Download Data Set S4, XLSX file, 0.3 MB. Copyright © 2016 Moriel et al. 2016 Moriel et al. This content is distributed under the terms of the Creative Commons Attribution 4.0 International license.

In order to define the *E. coli* accessory genome, 298,563 annotated ORFs (which comprised 290,776 chromosomal ORFs and 7,787 plasmid ORFs) from the 62 completely sequenced strains were compared to each other. This pairwise comparison identified 12,722 unique ORFs based on >75% protein identity that displayed a prevalence of <99% in EcoDS (10,513 chromosomal and 2,209 plasmid [see Data Set S5 in the supplemental material). While we accept that our arbitrary cutoff of >75% protein identity may result in the overlooking of some biological variation, as previously described in *Shigella* (24) and EPEC (25), our analysis nevertheless reflects the enormous genetic diversity that exists in the pan-*E. coli* genome. We refer to this gene set as the accessory *E. coli* genome. We note that no genes absent in MG1655 exhibited >99% prevalence in EcoDS, thus providing support to our overall determination of the core genome (1,037 genes) and the conserved subset of *E. coli* genes (3,042 genes).

10.1128/mSphere.00326-16.7 Data Set S5  The *E. coli* accessory genome. Download Data Set S5, XLS file, 2.6 MB. Copyright © 2016 Moriel et al. 2016 Moriel et al. This content is distributed under the terms of the Creative Commons Attribution 4.0 International license.

### Identification of novel vaccine targets.

The definition of a conserved set of 3,042 *E. coli* genes provides a framework for the development of new tools in epidemiology, diagnostics, and vaccine antigen discovery. In order to evaluate the expression of core components as potential antigens that could be targeted for vaccine development, we examined the proteome of strain EC958, which belongs to a clinically relevant and globally disseminated MDR sequence type (ST131). Proteomic analysis of outer membrane vesicles (OMVs) induced from EC958 led to the identification of 115 proteins (see Data Set S6 in the supplemental material). Among them, 23 were predicted to have an extracellular (*n* = 1), outer membrane (*n* = 15), or unknown (*n* = 7) subcellular localization. To refine this list, we focused our attention on proteins encoded by genes that were prevalent in more than 99% of EcoDS. This led to a panel of 17 potential surface-exposed antigens, which was even further reduced by removing outer membrane proteins predicted to have a transmembrane β-barrel structure based on analysis by PHYRE2 (i.e., OmpA, OmpC, OmpF, OmpX, MipA, Fiu, FepA, Tsx, and CirA) and proteins with an unknown subcellular localization and no predicted signal sequence based on LipoP (WrbA, SodA, TrmL, and LysC). This left a final list of four potential surface-associated proteins, namely, BamC, OsmE, SlyB, and YncE.

10.1128/mSphere.00326-16.8 Data Set S6  Proteomic profile of EC958 OMVs. Download Data Set S6, XLSX file, 0.1 MB. Copyright © 2016 Moriel et al. 2016 Moriel et al. This content is distributed under the terms of the Creative Commons Attribution 4.0 International license.

### YncE is a highly immunogenic and protective antigen.

**FIG 3:**
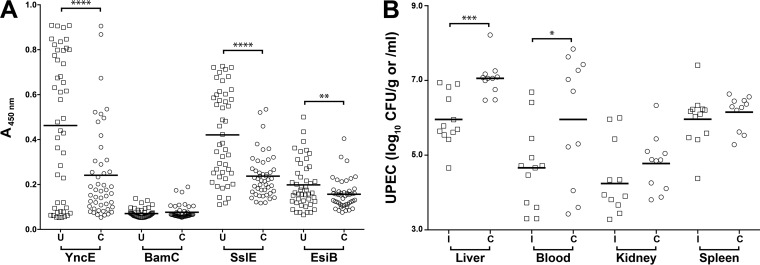
Immunoreactivity of plasma from urosepsis patients to *E. coli* vaccine antigens and bacterial burden following intravenous challenge in mice vaccinated with YncE. (A) Blood plasma was collected from 47 urosepsis patients (U) at least 4 days post-admittance to the hospital. IgG-specific antibody levels were compared to those from 47 healthy volunteers with no recent history of UTI (C). (B) Level of infection in immunized (I) and control (C) groups of mice at 24 h following intravenous challenge with UPEC strain CFT073. Symbols represent individual mice, and bars show the medians. The limit of detection was 200 CFU/g or CFU/ml. Statistically significant *P* values are indicated: ****, *P* < 0.0001; ***, *P* < 0.001; **, *P* < 0.01; *, *P* < 0.05.

To examine these four proteins further, each respective gene was PCR amplified and cloned in frame with an N-terminal 6×His tag sequence. Expression studies using these constructs revealed only the BamC and YncE proteins were produced as soluble recombinant proteins (see Fig. S2A in the supplemental material). Therefore, these two proteins were purified and tested for immunogenicity using plasma obtained from convalescent urosepsis patients and plasma from an age- and sex-matched healthy control group. As a positive control and correlate of immunogenicity, we also included SslE (ecok1_3385) and EsiB (c5321) in our analysis, both of which have previously been shown to be protective against extraintestinal pathogenic *E. coli* (ExPEC) infection in a mouse sepsis model (26). Both SslE (*P* < 0.0001) and EsiB (*P* < 0.01) showed higher reactivity to the plasma from urosepsis patients compared to healthy individuals (Fig. 3A). Among the targets identified in this study, BamC showed no significant reactivity with plasma from urosepsis patients. However, in contrast, YncE was strongly reactive with plasma from urosepsis patients compared to healthy individuals (*P* < 0.0001), suggesting it is expressed during human infection.

10.1128/mSphere.00326-16.2 Figure S2  (A) Expression of selected targets. TL, total cell lysates; SF, soluble fraction. (B) Murine YncE-specific IgG response following subcutaneous immunization. Median titers with the full range of variation are plotted in logarithmic scale. Download Figure S2, TIF file, 0.4 MB. Copyright © 2016 Moriel et al. 2016 Moriel et al. This content is distributed under the terms of the Creative Commons Attribution 4.0 International license.

We investigated the ability of YncE to elicit a protective immune response against acute systemic *E. coli* infection using an established murine model of bacteremia (27). YncE was highly immunogenic, inducing a strong IgG response (Fig. S2B), and mice immunized with YncE were significantly protected against infection, as evidenced by lower blood and liver *E. coli* loads following systemic challenge (Fig. 3B). Taken together, these data identify YncE as a novel, highly conserved and strongly immunogenic *E. coli* antigen that is able to provide protection against acute systemic infection when administered by vaccination.

### YncE is broadly expressed and secreted by different *E. coli* pathotypes.

**FIG 4:**
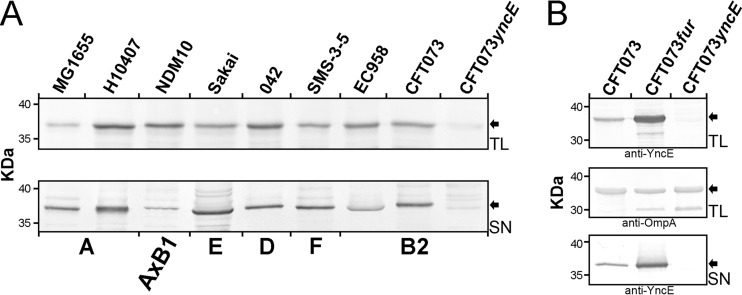
Western blot analysis of total cell lysate (TL) and supernatant (SN) fractions prepared from *E. coli* strains representing different phylogroups. (A) The YncE protein was detected in all TL and SN samples. The specificity of the antiserum was confirmed by the absence of a cross-reacting band in the CFT073 *yncE* mutant. (B) Overexpression of YncE in a *fur* knockout mutant. OmpA was used as an expression control.

The *yncE* gene was highly conserved among strains in EcoDS (*n* = 1,550/1,556 [99.6%]). Strains lacking the *yncE* gene were not related by ST or phylogroup: DEC13A, ST296/B1; DEC13B, ST296/B1; DEC13D, ST278/B1; IAI39, ST24/D; N1, ST515/B1; and UMEA 3687-1, ST73/B2. To verify the expression of YncE by strains belonging to different pathotypes and phylogroups, Western blot analysis was performed using rabbit polyclonal antibodies generated against recombinant YncE (Fig. 4A). It was observed that YncE is broadly expressed by *E. coli* strains from different pathotypes, including ETEC (strain H10407), EHEC (strain Sakai), EAEC (strain 42), and UPEC (strains NDM10, EC958, and CFT073). Moreover, Western blot analysis of strains representing the most predominant *E. coli* phylogroups and subgroups (A, AxB1, E, D, F, and B2) determined in this study revealed the presence of YncE in the supernatant after overnight growth (Fig. 4A). YncE was also overexpressed in an *E. coli fur* mutant during *in vitro* growth, demonstrating a possible role for iron in its regulatory control (Fig. 4B).

## DISCUSSION

Current vaccination strategies against *E. coli* have focused on individual pathotypes and targeted major virulence determinants. Examples include colonization factor antigens and heat-labile toxin from ETEC (28), components and effectors of the type III secretion system from EPEC (29), Shiga toxin from EHEC (30), and fimbrial adhesins and siderophore receptors from UPEC (31). In this study, we used an essential gene strategy to generate a curated data set of *E. coli* genomes (EcoDS) and used this diverse collection (representing 435 STs) to define core and accessory elements of the pan-*E. coli* genome. This information, together with proteomic data, led to the identification and validation of YncE as a highly conserved and protective *E. coli* vaccine antigen.

We observed that the clustering of different strains according to the seven housekeeping MLST genes was consistent with a modern framework composed of phylogroups A, AxB1, B2, and D (6). Moreover, we observed that single subpopulations comprehended strains involved in multiple diseases, supporting previous studies that demonstrated strains from the same pathotype can be distributed over the entire span of phylogenetic diversity and are not restricted to one specific phylogroup (5–7). Therefore, despite the high diversity, complexity, and low prevalence of the accessory elements in EcoDS, *E. coli* strains possess a defined set of highly prevalent genes irrespective of pathotype, phylogroup, and associated clinical disease.

Comparative analysis of the genomes in EcoDS showed that genes comprising the *E. coli* accessory genome were highly variable in prevalence. The accessory genome is considered a flexible gene pool shaped by mobile genetic elements and represents a major driver of *E. coli* evolution (32). In this study, we observed that the *E. coli* accessory genome comprises a broad set of 12,722 ORFs (prevalence of <99% in EcoDS) and that approximately 90% of the entire accessory genome is present in less than 90% of strains in EcoDS, which poses an enormous challenge for broad therapeutic interventions. Potential *E. coli* universal vaccine targets such as FimH and SslE, for example, were present in approximately 89% and 70% of the strains in EcoDS, respectively.

A smaller subset of 3,042 genes present in more than 99% of strains in EcoDS was also defined. These genes were used to trace novel vaccinology strategies. In order to identify potential surface-exposed and secreted proteins from the *E. coli* core genome that could represent new vaccine targets, we investigated the OMV-associated proteome of the MDR ST131 strain EC958. Although OMVs are not only represented by surface-exposed proteins, its combination with *in silico* subcellular localization prediction tools can facilitate the identification of exposed targets (33). Among the 115 proteins identified, we selected four proteins for cloning and expression based on literature analysis, predicted subcellular localization, and structural conformation. Among them, YncE showed increased reactivity to a plasma collection of urosepsis convalescent patients, higher than the titers obtained for SslE, a type II secreted mucinase originally identified from ExPEC (26, 34) that provides broad protection against *E. coli* infection in different animal models (34). BamC, a surface-associated lipoprotein (35), showed no significant reactivity with the urosepsis plasma collection. Moreover, YncE was also shown to be highly immunogenic and decreased the level of infection in a murine model of bacteremia, confirming the immunogenicity of this antigen and its potential as a broad vaccine candidate against *E. coli*.

YncE is a seven-bladed beta-propeller (36) transported by the Sec machinery (37) and associated with binding to single-stranded DNA (38). We have previously shown that YncE is present in the OMV proteome of a large collection of urosepsis strains (39), and in this study, we demonstrated that YncE is present in the OMV proteome of EC958 (phylogenetic group B2) and in the secretome of strains representing all other phylogenetic groups. Moreover, we confirmed the regulation of YncE by Fur (40), indicating its potential role during infection in iron-limiting environments such as blood and the urinary tract. Taken together, our results indicate that YncE fulfills many prerequisites required for a vaccine candidate: YncE is (i) immunogenic, (ii) highly prevalent, (iii) highly conserved, (iv) soluble, (v) stable, and (vi) expressed during infection.

In conclusion, we have demonstrated the genome complexity and plasticity of *E. coli* and dissected the difficulty associated with targeting the accessory genome for broadly therapeutic interventions. Moreover, we confirmed the close association between different pathogenic and nonpathogenic *E. coli* lineages. We also designed a strategy based on the *E. coli* core genome for the identification of novel potential vaccine targets, which led to the discovery of YncE as an immunogenic and protective antigen. Although we cannot predict the impact of this conserved antigen to the microbiome, recent studies have shown that vaccination with conserved antigens indicate no significant interference with the microbiome (41). Even though YncE is a highly prevalent vaccine antigen, we envisage its use in combination with pathotype-specific antigens rather than in standalone formulations, which would also help to define a target population for clinical testing. Additional work is now required to evaluate the efficacy of YncE immunization in different animal infection studies, alone and in combination with other broadly prevalent or pathotype-specific antigens.

## MATERIALS AND METHODS

### The *E. coli* data set and MLST analysis.

The *E. coli* database was represented by 62 complete and 1,638 draft genomes available on the NCBI public database as of 1 January 2014 (see Data Set S7 in the supplemental material).

10.1128/mSphere.00326-16.9 Data Set S7  The 1,700 *E. coli* genome sequences used in this study. Download Data Set S7, XLSX file, 0.1 MB. Copyright © 2016 Moriel et al. 2016 Moriel et al. This content is distributed under the terms of the Creative Commons Attribution 4.0 International license.

### Bioinformatic analysis.

Sequence comparisons were performed using the FASTA36 package (42). Subcellular localization was predicted by PSORTb 3.0 (43), and signal sequence was predicted by LipoP 1.0 (44). Structural analysis was performed by PHYRE2 (45). The core and accessory genome was determined by amino acid sequence identity using tfastx36 (42) and a cutoff of >75% over a 75% alignment. Strains were classified into the major A, B1, B2, and D phylogroups using an *in silico* triplex analysis of the *chuA*, *yjaA*, and TSPE4.C2 loci (46, 47); analysis was performed by nucleotide sequence identity using FASTA36 (42) and a cutoff of >90% over a 90% alignment. Further *in silico* classification of selected strains into other less common phylogroups was performed using an extension of this scheme (48). MLST analysis was performed using the sequence of seven housekeeping genes as previously described (8). Phylogenetic trees were drawn using MEGA6 (49) using the concatenated sequence of the seven housekeeping genes. Circular representations were drawn using Circos (50).

### Bacteria and growth conditions.

*E. coli* strains were routinely grown at 37°C on solid or in liquid Luria-Bertani (LB) medium supplemented with the appropriate antibiotics: chloramphenicol (30 µg/ml), kanamycin (50 µg/ml), and ampicillin (100 µg/ml). For the generation of total cell lysates and supernatant fractions, bacteria were inoculated in liquid LB to a starting optical density at 600 nm (OD_600_) equal to 0.050, and cultures were grown overnight at 37°C with shaking (180 rpm). Cells were harvested at 10,000 × *g* for 10 min at 4°C to generate the whole-cell lysate sample. Supernatant fractions were generated as previously described (51). To obtain EDTA-treated heat-induced OMVs, *E. coli* EC958 was grown in minimal M9 medium supplemented with Casamino Acids at 37°C under shaking conditions (180 rpm) and harvested at a final OD_600_ of 0.5. Cells were centrifuged at 10,000 × *g* for 10 min at 4°C, and the pellet was used for OMV heat induction.

### Molecular methods, proteomic analyses, and immunoblotting.

Genomic DNA was extracted using the Wizard Genomic DNA purification kit (Promega). PCR was performed using Phusion High-Fidelity DNA polymerase (New England Labs) and primers described in Data Set S8 in the supplemental material. PCR products were purified from agarose gel using the QIAquick gel extraction kit (Qiagen) and cloned into pMCSG7 using the ligation-independent cloning (LIC) method (52). DNA was transformed into chemically competent *E. coli* TOP10 cells (Invitrogen) for plasmid propagation. The primers used for cloning are described in Data Set S8. Isolation of plasmid DNA was carried out using the QIAprep spin miniprep kit (Qiagen), and DNA was sequenced using the BigDye Terminator v3.1 cycle sequencing kit (Invitrogen). Correct constructs were used to transform *E. coli* BL21(DE3)(pLysS) (Invitrogen) for protein expression. A CFT073 *yncE* mutant was constructed by λ-Red mediated homologous recombination using primers 5600 and 5601 (Data Set S8) as previously described (53). The formation of OMVs was induced by incubation in 0.01 M EDTA at 56°C, and samples were analyzed by high-performance liquid chromatography tandem mass spectrometry (HPLC-MS/MS) as previously described (39, 51). The peptide fingerprint was evaluated using ProteinPilot software 4.0 in combination with the EC958 protein database. SDS-PAGE and immunoblotting were performed as previously described (54) using a YncE-specific antibody. Rabbit YncE antibodies were raised against purified YncE by the Walter and Eliza Hall Institute Antibody Facility as previously described (55).

10.1128/mSphere.00326-16.10 Data Set S8  Primers used in this study. Download Data Set S8, XLSX file, 0.1 MB. Copyright © 2016 Moriel et al. 2016 Moriel et al. This content is distributed under the terms of the Creative Commons Attribution 4.0 International license.

### Expression and purification of vaccine targets.

Polyhistidine-tagged recombinant proteins were obtained by autoinduction (56) and purified by affinity and gel filtration chromatography. Briefly, BL21(DE3)(pLysS) cells harboring the LIC vectors were grown in ZYP-5052 (1 liter) overnight at 28°C at 250 rpm. Cells were harvested by centrifugation at 18,600 × *g*, resuspended in a mixture of 25 mM Tris-HCl (pH 7), 150 mM NaCl, 0.5% Triton X-100, protease inhibitor cocktail (Sigma), and DNase I (Roche), and lysed by sonication (Misonix, Inc., XL-2000; QSonica). Cell debris was removed by centrifugation, and histidine-tagged proteins were purified using Talon metal affinity resin (Clontech) preequilibrated with buffer A (25 mM HEPES [pH 7], 150 mM NaCl). Proteins bound to the resin were washed extensively with buffer B (25 mM Tris-HCl, 500 mM NaCl, 10 mM imidazole) and eluted with buffer C (25 mM Tris-HCl [pH 7], 150 mM NaCl, 250 mM imidazole). Fractions containing the eluted protein were pooled and dialyzed overnight in buffer A and further purified by gel filtration chromatography (Äkta, GE Healthcare) using a HiLoad 16/60 Superdex 75 prep-grade column (GE Healthcare) preequilibrated in buffer A.

### Plasma collections and immunoassays.

Blood plasma was collected from 47 urosepsis patients admitted to the Princess Alexandra Hospital (Brisbane, Australia) and 42 healthy volunteers with no recent history of UTI. Recombinant proteins (10 µg/ml) were coated onto Nunc Maxisorp flat-bottom 96-well plates (Thermo Scientific) in carbonate coating buffer (18 mM Na_2_CO_3_, 450 mM NaHCO_3_ [pH 9.3]) at 4°C overnight. Plates were then washed twice with phosphate-buffered saline–0.05% Tween 20 (PBST) and blocked on 5% skim milk in PBST (150 µl) for 90 min at 37°C. Plates were washed four times with PBST, and then plasma samples were added to the wells at a 1:10 dilution. Plates were incubated for 90 min at 37°C and washed four times with PBST. Peroxidase-conjugated anti-human IgG (1:30,000 dilution in 0.5% skim milk) was applied as a secondary antibody and incubated for 90 min at 37°C. Plates were washed four times with PBST before undergoing development with 3,3′,5,5′-tetramethylbenzidine. Reactions were stopped with 1 M HCl. Intensity was determined using SpectraMax 190 absorbance microplate reader at 450 nm. Statistical analysis for comparisons between patient and healthy plasma was performed using an unpaired two-sample *t* test. A statistical significance threshold was set at *P* < 0.05.

A rabbit polyclonal antiserum was raised against purified YncE using four immunizations (400 µg recombinant protein/dose) at the Walter and Eliza Hall Institute Antibody Facility. For immunoblotting, samples were subjected to SDS-PAGE using 12% Bis-Tris gels and subsequently transferred to polyvinylidene difluoride (PVDF) microporous membrane. YncE antiserum was used as primary serum, and the secondary antibody was alkaline phosphatase-conjugated anti-rabbit IgG. Sigma Fast 5-bromo-4-chloro-3-indolylphosphate–nitroblue tetrazolium (BCIP/NBT) was used as the substrate in the detection process.

### Bacteremia model of infection.

A murine model of *E. coli* bacteremia, as previously described (27), was used to assess the protective efficacy of YncE as a vaccine immunogen against systemic infection. Groups of 12 C57BL/6 mice (8 to 12 weeks old) were immunized subcutaneously (s.c.) with 100 µg of YncE in 100 µl of an emulsification of PBS and complete Freund’s adjuvant (Sigma) (2:1) on day 0. Booster doses of 25 µg of antigen in 100 µl of an emulsification of PBS and incomplete Freund’s adjuvant (Sigma) (1:1) were administered s.c. on days 7 and 14, essentially as previously described (57). Mice were challenged intravenously (i.v.) with approximately 6.4 × 10^6^ CFU of *E. coli* CFT073 in 200 µl of PBS via the lateral tail vein on day 21. The burden of disease was assessed at 24 h postchallenge by quantitating the bacterial loads in liver, blood, kidney, and spleen. The experiment included mock-immunized controls (which received PBS and adjuvant only) and was repeated independently.

Blood samples were also collected on days 0, 7, 14, 21, and 22 to measure YncE-specific IgG antibody titers by enzyme-linked immunosorbent assay (ELISA). Sera from immunized mice were separated from blood at 1,500 × *g* for 10 min. Plates were coated, blocked, and washed as detailed above. Mouse serum samples were applied at a 1:2 serial dilution, starting from a 1:10 dilution. Subsequent wash, secondary antibody, and development steps were performed as previously described (54), with the exception that peroxidase-conjugated anti-mouse antibodies were applied as the secondary antibody. YncE IgG titers were defined as the logarithmic dilution that produced a significant absorbance (450 nm) in comparison to that of a blank sample.

### Ethics statement.

This study was performed in accordance with the ethical standards of the University of Queensland, Princess Alexandra Hospital, Grifﬁth University, and the Helsinki Declaration. The collection of human plasma was approved, and the need for informed consent was waived, by the institutional review boards of the Princess Alexandra Hospital (research protocol 2008/264) and Grifﬁth University (MSC/18/10/HREC). Approval for mouse infection studies was obtained from The Griffith University Animal Ethics Committee (MSC/03/15/AEC).
