# Supplementary figures and images for: A Novel Protective Vaccine Antigen from the Core Escherichia coli Genome
Source: mSphere. 2016 Nov 23;1(6):e00326-16. doi: 10.1128/mSphere.00326-16 (PMC5120174; doi:10.1128/mSphere.00326-16)

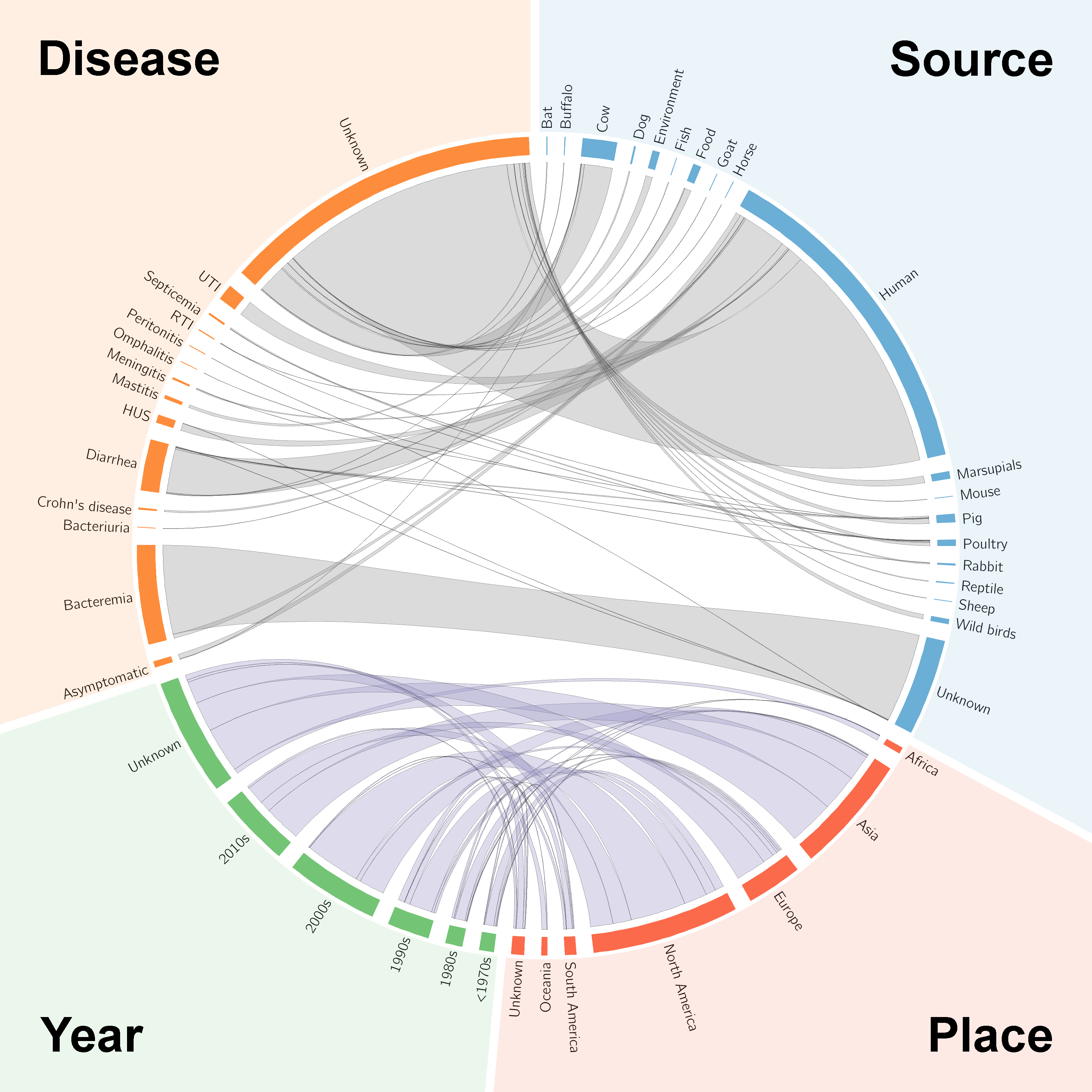

Supplement: Figure S1 [file sph006162191sf1.tif]

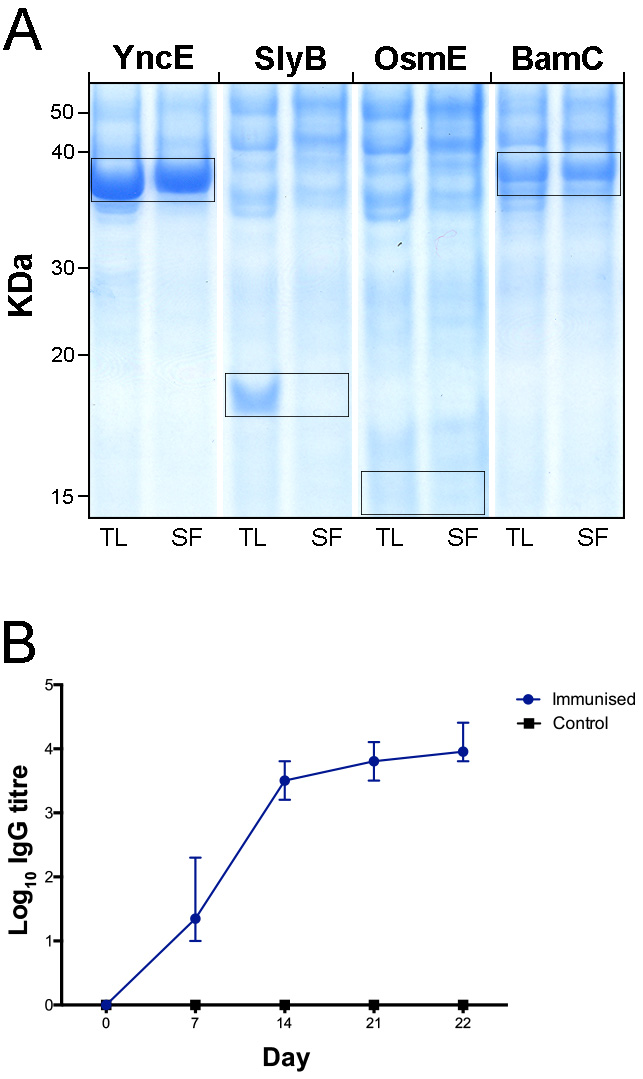

Supplement: Figure S2 [file sph006162191sf2.tif]
